# Genetic diversity of VIR Raphanus sativus L. collections on aluminum tolerance

**DOI:** 10.18699/VJ20.655

**Published:** 2020-10

**Authors:** A.B. Kurina, I.A. Kosareva, A.M. Artemyeva

**Affiliations:** Federal Research Center the N.I. Vavilov All-Russian Institute of Plant Genetic Resources (VIR), St. Petersburg, Russia; Federal Research Center the N.I. Vavilov All-Russian Institute of Plant Genetic Resources (VIR), St. Petersburg, Russia; Federal Research Center the N.I. Vavilov All-Russian Institute of Plant Genetic Resources (VIR), St. Petersburg, Russia

**Keywords:** radish and small radish, collection, genetic diversity, acidic soils, eriochrome cyanine R, early diagnosis, aluminum resistance, коллекция редиса и редьки, генетическое разнообразие, кислые почвы, эриохромцианин, ранняя диагностика, алюмоустойчивость

## Abstract

Radish and small radish (Raphanus sativus L.) are popular and widely cultivated root vegetables in the world, which occupy an important place in human nutrition. Edaphic stressors have a significant impact on their productivity and quality. The main factor determining the phytotoxicity of acidic soils is the increased concentration of mobile aluminum ions in the soil solution. The accumulation of aluminum in root tissues disrupts the processes of cell division, initiation and growth of the lateral roots, the supply of plants with minerals and water. The study of intraspecific variation in aluminum resistance of R. sativus is an important stage for the breeding of these crops. The purpose of this work was to study the genetic diversity of R. sativus crops including 109 accessions of small radish and radish of various ecological and geographical origin, belonging to 23 types, 14 varieties of European, Chinese and Japanese subspecies on aluminum tolerance. In the absence of a rapid assessment methodology specialized for the species studied, a method is used to assess the aluminum resistance of cereals using an eriochrome cyanine R dye, which is based on the recovery or absence of restoration of mitotic activity of the seedlings roots subjected to shock exposure to aluminum. The effect of various concentrations on the vital activity of plants was revealed: a 66-mM concentration of AlCl_3_ · 6Н_2_О had a weak toxic effect on R. sativus accessions slowing down root growth; 83 mM contributed to a large differentiation of the small radish accessions and to a lesser extent for radish; 99 mM inhibited further root growth in 13.0 % of small radish accessions and in 7.3 % of radish and had a highly damaging effect. AlCl_3_ · 6Н_2_О at a concentration of 99 mM allowed us to identify the most tolerant small radish and radish accessions that originate from countries with a wide distribution of acidic soils. In a result, it was possible to determine the intraspecific variability of small radish and radish plants in the early stages of vegetation and to identify genotypes that are contrasting in their resistance to aluminum. We recommend the AlCl_3_ · 6Н_2_О concentration of 83 mM for screening the aluminum resistance of small radish and 99 mM for radish. The modified method that we developed is proposed as a rapid diagnosis of aluminum tolerance for the screening of a wide range of R. sativus genotypes and a subsequent study of contrasting forms during a longer cultivation of plants in hydroponic culture (including elemental analysis of roots and shoots, contrasting in resistance of accessions) as well as reactions of plants in soil conditions.

## Abstract (ru)

Редис и редька (Raphanus sativus L.) – популярные и широко возделываемые в мире корнеплод- ные овощные культуры, которые занимают важное место в питании человека. На их продуктивность и каче- ство существенное влияние оказывают эдафические стрессоры. Основным фактором, определяющим фи- тотоксичность кислых почв, служит повышенная концентрация подвижных ионов алюминия в почвенном растворе. Аккумуляция алюминия в тканях корня нарушает процессы деления клеток, инициации и роста боковых корней, снабжения растения минеральными веществами и водой. Изучение внутривидовой измен- чивости по алюмоустойчивости R. sativus является важным этапом в селекции этих культур. Цель настоящего исследования заключалась в изучении генетического разнообразия культур R. sativus на примере 109 образ- цов редиса и редьки различного эколого-географического происхождения, принадлежащих 23 сортотипам, 14 разновидностям европейского, китайского и японского подвидов, по признаку устойчивости к токсическому действию ионов алюминия. При отсутствии специализированной для вида методики экспресс-оценки взят метод оценки алюмоустойчивости с использованием эриохромцианинового красителя, разработанный для зерновых культур, в основе которого лежит учет степени восстановления митотической активности кор- ней проростков, подвергнутых шоковому воздействию повышенных концентраций алюминия. Выявлено влияние различных концентраций на жизнедеятельность растений: концентрация хлорида алюминия 66 мМ оказывала слабое токсическое действие на образцы R. sativus, замедляя отрастание корней; концентрация 83 мМ оказалась в высокой степени дифференцирующей для редиса и в меньшей – для редьки; концентра- ция 99 мМ полностью ингибировала дальнейший рост корней у 13.0 % образцов редиса и 7.3 % редьки и обладала высоко повреждающим эффектом. Концентрация AlCl_3_ · 6Н_2_О 99 мМ позволила выделить наиболее высокотолерантные образцы редиса и редьки, которые происходят из стран с широким распространением кислых почв. В результате исследований широкого разнообразия мировой коллекции определена внутри- видовая изменчивость редиса и редьки на ранних этапах вегетации и идентифицированы контрастные по устойчивости к алюминию генотипы. Мы рекомендуем концентрацию 83 мМ AlCl_3_ · 6Н_2_О для скрининга алю- моустойчивости образцов редиса, а концентрацию 99 мМ – для образцов редьки. Разработанный нами мо- дифицированный метод предлагается в качестве экспресс-диагностики алюмотолерантности для быстрого скрининга широкого спектра генотипов R. sativus и последующего изучения контрастных форм при более длительном выращивании растений в гидропонной культуре (включая элементный анализ корней и побе- гов, контрастных по устойчивости образцов), а также реакций растений в почвенных условиях.

## Introduction

Aluminum is one of the most abundant metals in the earth’ crust (Fitzpatrick, 1986; Kochian et al., 2015) and is considered non-toxic to plants when the soil solution is neutral or slightly alkaline. Natural processes or human activities can lead to an increase acidity in soil, in result of which the solubility of aluminum increases, and the content of its mobile forms (Al^3+^) increases (Lin-Tong et al., 2013), that makes aluminum the main toxic factor in acidic soils (Klimashevskiy, 1991; Kochian et al., 2004). Acidic soils in the world make up 30–40 % of arable ground and up to 70 % of ground that can potentially be used as arable (Suhoverkova, 2015). In Russia in 2019, out of 50 million hectares of excessively acidic soils, strongly and moderately acidic ones occupy from 25 to 35 million hectares, which is about 30 % of all arable ground (Vorob’ev, 2019).

The toxicity of Al^3+^ ions reduces productivity by inhibiting root growth and affecting water and nutrient absorption. A number of studies have described the symptoms of aluminum poisoning associated with impaired permeability of the cell wall, plasma membrane, mitochondrial, cytoskeleton, and nuclear functions (McNeilly, 1982; Roy et al., 1988; Aniol, 1997; Kabata-Pendias, 2010). So, aluminum affects on a series of cellular processes, including the rate of cell division, and disrupts the properties of protoplasm and cell walls.

Plants are subdivided into resistant and sensitive by alumotoxicity, varietal differences may be stronger than species (Hanson, Kamprath, 1979; Klimashevskiy, 1991). Plants have developed several mechanisms of resistance to aluminum during the evolutionary process (Kochian et al., 2005, 2015; Ma, 2007; Ma et al., 2014). In recent years, the molecular mechanism of aluminum tolerance in agricultural crops, primarily in cereals, has been actively studied (Liu et al., 2014; Ma et al., 2014; Kochian et al., 2015). Significant progress has been achieved in understanding the physiological and molecular mechanisms of aluminum tolerance in Arabidopsis (Hoekenga et al., 2006), rapeseed (Ligaba et al., 2006), maize (Ligaba et al., 2012), soybean (Peng et al., 2018), rice (Huang et al., 2012; Che et al., 2018), sorghum (Huang et al., 2018; Melo et al., 2019), rye (Collins et al., 2008; Yokosho et al., 2010) and wheat (Gruber et al., 2010; Wang et al., 2015).

At present, aluminum resistance is considered as a complex phytoecological problem, from the solution of which an obtaining of guaranteed productivity crops on acidic soils depends. The identification of genes and mechanisms of aluminum tolerance makes possible Al-tolerant species and cultivars of agricultural crops breeding using molecular and transgenic approaches (Delhaize et al., 2004; Magalhaes et al., 2007; Pereira et al., 2010).

The basic critical parameter for the successful creation of stress tolerant cultivars is the genetic diversity of the initial material for this indicator as a material for selection (Lisitsyn, Amunova, 2014). The successful creation of aluminum-resistant cultivars of agricultural plants is based on a significant variability in the trait of aluminum tolerance and relatively simple methods of screening and breeding (Batalova, Lisitsyn, 2002; Kosareva, 2012). The search for genotypes with a high tolerance to Al is of great importance for agriculture on acidic soils.

Radish and small radish belong to the species Raphanus sativus L., for which two primary geographical centers of origin are known – Mediterranean and Asian (Vavilov, 1965), herewith the Asian center was divided into secondary centers in the classification of M.A. Shebalina and L.V. Sazonova (1985): South West Asian, East Asian, South Asian tropical. Small radish is a mutant form of radish; artificial selection was carried out on the feature of dwarfishness of plants in the vegetative period of ontogenesis, while the plants of the reproductive period practically do not differ in habitus from the radish plants. The processes of mutagenesis in R. sativus are determined by the climatic conditions of the places of origin of cultural forms. Cultivation of radish began 4–3 thousand BC, small radish was introduced into culture much later – the first information about it appeared in Italy at the beginning of the 16th century.

Small radish cultivars are assigned to 6 botanical varieties and 16 types, radish – 14 varieties and 20 types, which differ in a complex of morphological, phenological, physiological, biochemical and economically valuable traits. Small radish and radish are popular and widely cultivated root vegetable crops around the world that play an important role in human nutrition. They are valued for their high productivity, manufacturability, good taste and valuable biochemical composition.

For the growth and development of small radish and radish, the neutral reaction of the soil solution (pH 6.0–8.0) is the favorable. Plants are especially sensitive to low acidity in the initial periods of growth. Most of the spaces under small radish and radish in the world are located on the territory occupied by acidic soils; alumotoxicity makes a negative contribution to the decrease of the productivity and quality of these crops. Therefore, modern cultivars have to be tolerant to Al, alongside with signs of high productivity, resistance to pathogens, manufacturability, etc. The first stage in such studies should be the search in the gene pool of R. sativus for forms resistant to aluminum in an acidic environment.

Several diagnostic methods have been used to assess the degree of plant resistance to aluminum (Kosareva et al., 1995). Often used laboratory screening techniques are based on various modifications of methods for germinating seeds in an aquatic culture in the presence of toxic aluminum concentrations (Foy, 1996; Lisitsyn, 1999; Gupta, Gaurav, 2014). The advantage of such techniques is the simplicity of execution, low time spent, high throughput, and the ability to diagnose genotypes at the early stages of ontogenesis. A series of studies revealed a quite high correlation (r = 0.71…0.85) between the results of laboratory assessments of resistance at the early stages of development with the data of field and vegetation tests of adult plants (Aniol, 1981; Klimashevskiy, 1991; Baier et al., 1995; Burba et al., 1995).

Plant resistance can be assessed in laboratory tests by the degree of damage of the seedlings roots by aluminum using hematoxylin (Canado et al., 1999) and eriochrome cyanine R (Aniol, 1981). This method was successfully applied to assess the intraspecific variability of aluminum tolerance in rice (Awasthi et al., 2017), peas, maize, wheat, and sorghum (Anas, Yoshida, 2004; Kosareva, 2012; Vishnyakova et al., 2015) with hematoxylin, and with wheat, rye, triticale (Aniol, 1981; Aniol, Gustafson, 1984), aegilops, oats, maize (Kosareva, Semenova, 2004; Kosareva, 2012) and peas (Vishnyakova et al., 2015) with eriochrome cyanine R.

Researches of R. sativus root crops resistance to damage of aluminum have practically not been conducted. The toxicological effect of aluminum-based coagulants on various crops, including individual radish genotypes, was studied in the work of K. Zhang and Q. Zhou (2005). Oil radish (Raphanus sativus var. oleifera Metzg.) has the greatest potential for phytoextraction of fluorides from contaminated soils (Sokolova et al., 2019). J. Raj and L.R. Jeyanthi (2014) studied the effect of aluminum chloride on the germination of R. sativus seeds, and it was found that the maximum allowable limit for Al to maintain viability is 10 mM. The study of intraspecific variation of R. sativus aluminum resistance is an important stage for the breeding of these crops.

The purpose of this work was to study the genetic diversity of the VIR world wide R. sativus collection on the aluminum tolerance trait. The tasks were to determine the toxic concentration of aluminum chloride (AlCl_3_ · 6H_2_O), which differentiates small radish and radish accessions according to the degree of aluminum resistance, to identify the most resistant genotypes, and to determine their botanical, agrobiological, and geographic confinedness.

## Materials and methods

The object of research is the VIR core collections of small radish and radish, consisting of accessions of various ecological and geographical origin and most fully characterizing the diversity of the species.

The studied collection of small radish is represented by 54 accessions from 25 countries belonging to 13 cultivar types, 6 varieties of European and Chinese subspecies. The collection of radish is represented by 55 accessions from 17 countries, belonging to 10 cultivar types, 8 varieties of European, Chinese and Japanese subspecies (see the Table).

**Table 1.**
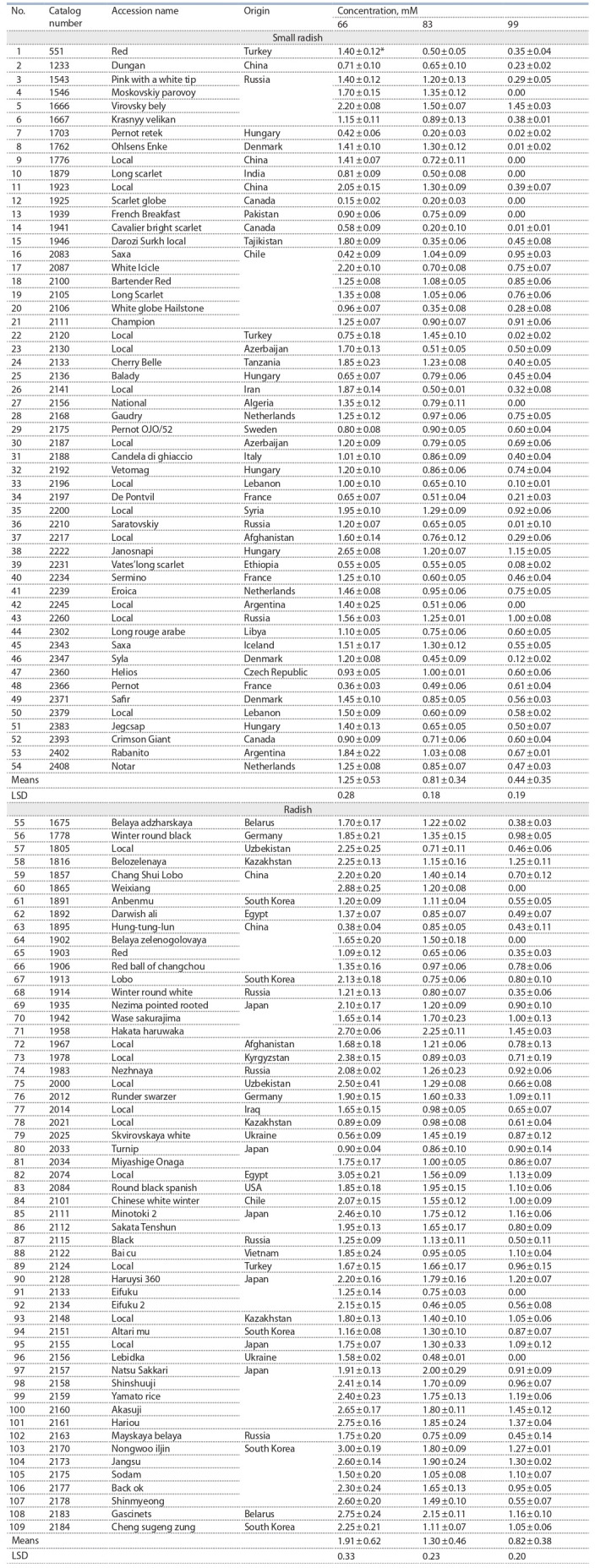
Characterization of R. sativus accessions along the length of root growth at various concentrations of aluminum chloride * MEAN ± SD.

In the absence of a rapid assessment methodology specialized for the studied species, the method of the aluminum resistance evaluation of cereals using an eriochrome cyanine R dye is used (Aniol, 1981), which is based on the recovery or absence of restoration of the seedlings roots mitotic activity subjected to shock exposure to aluminum.

The experiments were carried out in a climatic chamber with an illumination 7000 Lx, a temperature 19–21 °C and a photoperiod 16 h. Seeds (50 pieces of each accession) were placed in special cells for seeds and a mesh bottom, which were placed in 6-liter containers, placing them on the surface of the nutrient solution. The nutrient solution contained (mM): 0.4 CaCl_2_, 0.4 KNO_3_, 0.25 MgCl_2_, 0.01 (NH_4_)_2_SO_4_, 0.04 NH_4_NO_3_; pH 4.2 (Aniol, Gustafson, 1984). After germinating the seeds for 3 days, the not viable ones were rejected. Then, the cuvettes with seedlings were placed in a freshly prepared nutrient solution supplemented with aluminum chloride (AlCl_3_ · 6H_2_O) and incubated for 24 h.

Thus there are no descriptions of the R. sativus crops aluminum resistance in the publications, based on the preliminary experiments, we used AlCl_3_ · 6H_2_O concentrations of 66, 83, and 99 mM, which had a toxic effect on plants and inhibited root growth in degrees under the used conditions. After that, the cuvettes were placed in a fresh nutrient solution without aluminum and incubated for 48 h. During the indicated time, reparation processes took place in the roots (restoration of the mitotic activity of cells) and the roots grew. The seedlings were washed with clean water and the roots were stained by immersing the cuvettes in a 0.1 % solution of eriochrome cyanine R for 10 min. The excess dye was washed off with clean water, and the roots were dried with filter paper. The zone of root tissue damage with aluminum was colored violet after staining with eriochrome cyanine R. Plant resistance to aluminum was determined by the length of root tip regrowth. For each accession two independent experiments were carried out in two-fold repetition.

Statistical data processing was performed by the method of analysis of variance using the STATISTICA v.12.0 program (StatSoft Inc., USA), by the method of cluster analysis (Ward’s method) using the PAST program (Hammer et al., 2001).

## Results

At the first stage, we investigated the effect of different aluminum concentrations on small radish and radish. In general, the results of our research have shown that an excess of aluminum and hydrogen (low pH) in the nutrient solution negatively affects the growth and development of the embryonic roots of small radish and radish seedlings. We observed significant differences between R. sativus accessions in root regrowth at all tested concentrations of AlCl_3_ · 6H_2_O (see the Table).

The aluminum chloride concentration of 66 mM had a weak toxic effect on R. sativus accessions. In most of the small radish and radish accessions, the mitotic activity of seedling root cells was restored after the shock exposure to aluminum. In 70.4 % of the small radish accessions and 92.7 % of the radish, the root growth was rather high (more than 1.0 cm), that indicates a normal further development. 22.2 % of the small radish accessions and 5.5 % of the radish showed an average root growth (0.5–1.0 cm); in four small radish accessions and one radish, the root growth was less than 0.5 cm.

At a concentration of AlCl_3_ · 6H_2_O of 83 mM, a large differentiation of the accessions was observed. In 29.6 % of the small radish accessions and 70.9 % of the radish, the root growth was more than 1.0 cm, the average regrowth (0.5–1.0 cm) was observed in 51.9 % of the small radish and 25.5 % of the radish. Root growth of less than 0.5 cm was observed in 18.5 % small radish and 3.6 % radish accessions.

At an aluminum chloride concentration of 99 mM, there was no further root growth in 13.0 % of the small radish and in 7.3 % of the radish accessions. A slight root growth (up to 0.5 cm) was observed in 46.3 % of small radish and 14.5 % of radish. Root regrowth by 0.5–1.0 cm was observed in 33.3 % of small radish and 41.8 % of radish. Normal root growth after exposure of this concentration of the toxicant was observed in only 7.4 % of the small radish and 36.4 % of the radish accessions.

So, the differences were most clearly manifested between small radish accessions at Al concentration of 83 mM, and between radish accessions at a 99 mM concentration at different stressor intensity. These concentrations were used for further evaluation of polymorphism because their negative impact showed the maximum differentiating ability.

**Fig. 1.**
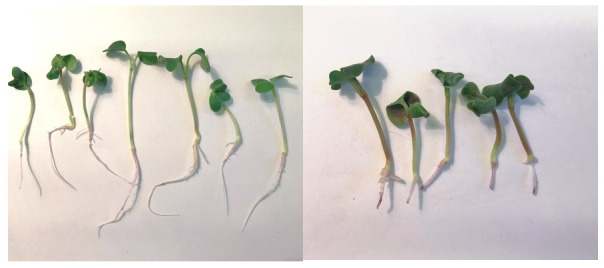
Appearance of resistant (left) and sensitive (right) small radish seedling.

The accessions with the minimum length of root regrowth had an intense violet coloration of the root areas that grew upon the addition of mobile aluminum, and the accessions with the maximum length of the root regrowth had a weak but detectable staining (Fig. 1).

**Fig. 2.**
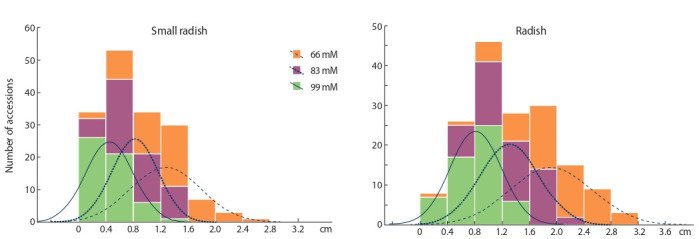
Histogram of the distribution of small radish and radish accessions along the length of root growth at various concentrations of aluminum chloride.

The accessions of small radish and radish were divided into several statistically significant groups according to the length of root regrowth, depending on the concentration of aluminum (Fig. 2). The accessions were characterized by a wide range of root growth at a concentration of 66 mM – 0.15–2.65 cm (small radish) and 0.38–3.05 cm (radish), this variability divided the samples into seven and eight groups, respectively.

Small radish accessions were divided at a concentration of 83 mM into four groups with a range of variability from 0.20 to 1.50 cm. The first group consisted of five accessions with root growth less than 0.40 cm; these accessions are of var. rubescens Sinsk. from Canada and Hungary. The second group included the largest number of accessions (24 accessions) from the countries of Minor Asia and Central Asia and Africa. The third group was represented by accessions of various types from Europe and South America. The fourth group included nine accessions with root regrowth more than 1.20 cm; these accessions are from Russia, China, Turkey, Hungary, Iceland, and Tanzania. Radish accessions were divided at a given concentration into five groups with a range of 0.46–2.25 cm. Accessions were absent with root regrowth after exposure to this concentration less than 0.40 cm. The first group included 8 accessions with root growth from 0.41 to 0.80 cm from Japan, Russia, China and Uzbekistan. The second group was represented by accessions from Central Asia, Vietnam, South Korea, Egypt and Japan. The third and fourth groups were the largest and included 31 accessions with root growth more than 1.20 cm from Japan, South Korea, countries of Europe and Central Asia, as well as from the USA, Chile and Russia. The fifth group was represented by 3 accessions from Japan and Belarus with root regrowth of more than 2.0 cm.

The small radish and radish accessions were divided at a concentration of 99 mM into four groups in the range from 0.00 to 1.45 cm. The first group consisted of 26 small radish accessions, of which 7 accessions did not have root regrowth; these accessions had different geographic origin, but most accessions were from Canada, Russia, China, and Central Asia. The first group of radish included only 7 accessions, of which four did not grow roots; this group included accessions from China, Ukraine, Belarus, and Russia. The second group of small radish was formed by accessions from Europe and South America, as well as some accessions from Azerbaijan, Tajikistan and Libya. This group of radish includes accessions from Russia, the countries of Central Asia, China and South Korea. The third group of small radish included 6 accessions from Chile, Russia and Syria, radish – 25 accessions mainly from Japan, South Korea, as well as from Chile, Turkey, Russia, Germany and the USA. The fourth group in small radish was formed by only one accession from Russia (k- 1666), in radish – 6 accessions from Japan, South Korea and Kazakhstan.

**Fig. 3.**
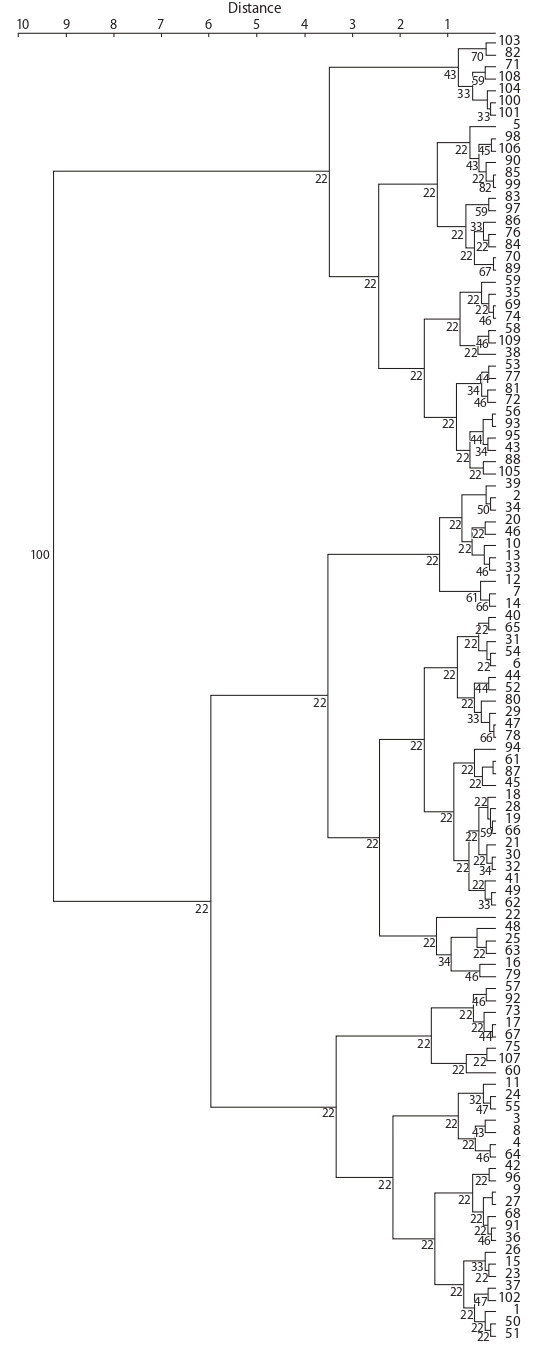
Dendrogram of R. sativus accessions by root growth after exposure to different concentrations of AlCl_3_ · 6H_2_O. Ward’s method. The numbers on the dendrogram indicate the size of the bootstrap. The numbers to the right of the dendrogram are accessions numbers in accordance with the Table.

Figure 3 shows a dendrogram based on the results of cluster analysis of root growth in R. sativus accessions after exposure to toxic concentrations of AlCl_3_ · 6H_2_O. According to the screening results using the Ward’s method, the small radish and radish accessions were divided into two big groups, each of the groups was divided into clusters according to the degree of aluminum resistance, the total number of which was five. The first group is represented by two clusters, the second – by three.

The first small cluster included accessions of Japanese radish from Japan and South Korea and Belarus and an accession of Chinese radish from Egypt; they showed a large root growth at a concentration of 66 mM AlCl_3_ · 6H_2_O and relatively high at concentrations of 83 and 99 mM. The second cluster combined accessions of small radish and radish with root growth more than 1.0 cm after exposure to all three toxic concentrations of Al. The cluster is divided into two subclusters. The first subcluster contains an accession of small radish from Russia (k-1666, Virovsky bely), accessions of Japanese radish from Japan and South Korea, two accessions of European winter radish from Germany and the USA (var. niger (L.) Sinsk.) and an accession of Chinese radish from Chile (var. lobo). The second subcluster includes accessions of small radish from Hungary (var. chloris Alef.), Syria (var. rubescens Sinsk.), Argentina (var. striatus Sinsk.) and Russia (var. roseus Sazon.), accessions of Chinese radish from Kazakhstan, China and South Korea (var. virens Sazon.), Japan, Russia, Iraq and Afghanistan (var. rubidus Sazon.) and accessions of Japanese radish from Japan, South Korea and Vietnam.

The third small cluster unites small radish accessions, which showed little or no root regrowth at all concentrations used. The cluster included accessions from France, Pakistan (var. striatus Sinsk.), Canada, Hungary, Ethiopia, Lebanon (var. rubescens Sinsk., var. radicula), China and India (var. roseus Sazon.).

The fourth cluster is represented by accessions of small radish and radish, in which root regrowth after exposure to toxic concentrations of 83 and 99 mM was average (up to 1.0 cm). The cluster is divided into three subclusters. The first subcluster included small radish accessions of var. striatus and var. rubescens, one accession each of var. radicula and var. roseus, Chinese radish from Kazakhstan (var. lobo) and China (var. roseus Sazon.) and Japanese radish from Japan. The second subcluster unites accessions of small radish from Chile, the Netherlands, Hungary (var. rubescens Sinsk.), accessions of European radish from Russia, Egypt (var. niger (L.) Sinsk.) and Chinese radish from South Korea (var. lobo), China (var. roseus Sazon.). The third subcluster includes accessions of small radish var. rubescens from Chile, Turkey and Hungary, accessions of European winter radish from Ukraine (var. hybernus), and an accession of pink Chinese radish from China.

The fifth cluster includes accessions of small radish and radish, with partial or complete inhibition of root growth at a concentration of 99 mM and an average root regrowth at other concentrations. The cluster is divided into three subclusters. The first subcluster unites accessions of Chinese radish of Central Asian origin, Japanese radish from Japan and South Korea, and an accession of small radish from Chile. The second subcluster mainly includes accessions of small radish from Russia, China and Tanzania and two accessions of radish from Belarus and China. The third subcluster is mainly represented by accessions of small radish of Central Asian origin and several accessions of radish from Russia and Ukraine.

## Discussion

Genetic processes were of great importance in the phylogenesis of radish and small radish: recombination, mutations at the chromosomal level, expression of inactive genes and changes in the frequencies of alleles that control traits and determine the phenotype of the plant; they occurred under natural and artificial selection in various ecological and geographical conditions (Bunin, Esikawa, 1993). The large intraspecific diversity of forms of R. sativus at the diploid level of development is explained by spontaneous gene and inherited somatic mutations (Campbell, Snow, 2009). In our previous studies, we found that the limits of variability of quantitative traits (morphological, productivity traits, early maturity, and accumulation of nutrients) in small radish and radish are very large (Kurina et al., 2017, 2018; Kurina, Artemyeva, 2017, 2019). For example, the amplitude of variation of the most important features: the duration of the period of vegetation is 18–95 days; root weight is 2–75 (small radish) and 150–1100 g (radish); the diameter of the leaf rosette is 8–45 cm; root shape: roundflat, round, round-oval, oval, cylindrical, fusiform, conical; content of ascorbic acid 18–55 mg/100 g, etc.

According to the literature data, it is known that, in general, small radish and radish are resistant to the action of heavy metals and have a high accumulating ability of heavy metals in the root (Wang et al., 2012; Ngo et al., 2016; Elizarieva et al., 2017). Japanese radish accumulates less toxic elements in roots; it is more resistant to pollution by such heavy metals as lead, cadmium, nickel, zinc, vanadium, chromium, arsenic. The response of Japanese radish to soil pollution is varietal specific (Gorelova et al., 2005; Xu et al., 2017). Crops of R. sativus are accumulators of heavy metals; they have been proposed for phytoremediation (Kumar et al., 1995; Ebbs, Kochian, 1997; Ebbs et al., 1997; Wang et al., 2012). Also, radish is a vegetable crop moderately sensitive to salt stress (Sun et al., 2016).

The study of R. sativus crops revealed high intraspecific variability in aluminum resistance. In general, radish was more resistant to alumo stress than small radish regardless of concentration, which is probably related to the processes of morphogenesis.

As a result of grouping accessions according to the length of root regrowth after exposure to various toxic concentrations of aluminum chloride (see Fig. 2), it was found that the accessions of both crops form four groups with a root regrowth range from 0 to 1.6 cm at a concentration of 83 and 99 mM. Accessions of R. sativus reacted weakly to low concentrations of AlCl3 · 6H2O, the mitotic activity of seedling root cells was restored after the shock effect of aluminum. With an increase of concentration, intraspecific differences in the crops begin to appear. The intensity of staining with eriochrome cyanine R characterizes the concentration of mobile forms of aluminum, which in turn correlates with aluminum tolerance (Vishnyakova et al., 2015). If, after treatment with aluminum, the concentration of its active forms is low, then the mitotic activity of cells is restored at the root, the root grows back, and after the staining zone, an unstained growth appears (Kosareva, 2012). So, the intensity of the staining can serve as an additional indicator of the degree of aluminum tolerance associated with the concentration of the toxicant in the root tissues.

Based on the obtained results, we propose a resistance scale for R. sativus crops based on aluminum tolerance: root growth up to 0.40 cm – sensitive, from 0.41 to 0.80 cm – weakly resistant, from 0.81 to 1.20 cm – medium resistant, more than 1.21 cm – highly resistant.

The AlCl_3_ · 6H_2_O concentration of 99 mM made possible to identify the most tolerant small radish samples (in descending order): Virovsky bely (k-1666, Russia), Janosnapi (k-2222, Hungary), Local (k-2260, Russia), and radish: Hakata haruwaka (k-1958, Japan), Akasuji (k-2160, Japan), Hariou (k-2161, Japan), Jangsu (k-2173, South Korea).

According to the results of cluster analysis, it was revealed that the first and second clusters combine highly resistant and medium-resistant radish accessions and highly resistant small radish accessions, the third cluster contains sensitive and low-resistant small radish accessions, and the fourth and fifth clusters mainly contain medium-resistant small radish accessions and low-resistant and unresistant radish accessions. It was revealed that accessions of R. sativus of Central Asian origin (Azerbaijan, Uzbekistan, Afghanistan, etc.), as well as from African countries (Algeria, Ethiopia) were found to be weak resistant and sensitive to alumostress. The soils of these countries are characterized by a neutral or slightly alkaline reaction of the soil solution, what, probably, determines the low resistance of the accessions to low acidity and alumostress. Medium-resistant accessions were mainly of European origin (Netherlands, Germany, Italy, etc.), as well as from the USA and Chile. In these countries, there is an active breeding of these crops in various directions. Accessions of small radish and radish from Russia, Hungary, Turkey, China, Japan, South Korea, and Kazakhstan had varying degrees of resistance; accessions of the same geographic origin could be both aluminum tolerant and sensitive to aluminum. Perhaps this is due to the presence of both acidic and neutral/alkaline soils in these countries, as well as to the direction of breeding work with these crops. The most aluminum-tolerant were accessions of Japanese radish from Japan of Kameido type and Shiroagiri type from South Korea, local accessions of green Chinese radish from Kazakhstan and accessions of Chinese small radish of the Russian breeding, which were obtained by selection and hybridization from the population of Asian radishes.

So, the Raphanus sativus species is polymorphic not only in phenotypic and biochemical characteristics, but also in the degree of resistance to various abiotic stresses.

## Conclusion

As a result of this study, we found that excess concentrations of mobile aluminum and hydrogen (elements of acidic soils) in the root zone lead to a negative effect on the growth and development of embryonic roots of small radish and radish accessions. In toxic concentrations of aluminum chloride in the nutrient medium, the accessions of the studied species were characterized by high variability in terms of aluminum tolerance at different stressor intensity. As a result of screening, we revealed the intraspecific variability of small radish and radish at the early stages of the growing season and identified genotypes contrasting in resistance to aluminum. We recommend a concentration of 83 mM AlCl_3_ · 6H_2_O for assessing the aluminum tolerance of small radish, and a concentration of 99 mM for assessing radish. The method developed by us is proposed as an express diagnostics of aluminum tolerance for rapid screening of a wide range of R. sativus genotypes and subsequent study of contrasting forms during longer plant cultivation in hydroponic culture (including elemental analysis of roots and shoots contrasting in the resistance of accessions), as well as plant reactions in soil conditions.

## Conflict of interest

The authors declare no conflict of interest.
